# High-Yield Production of Dihydroxyacetone from Crude Glycerol in Fed-Batch Cultures of *Gluconobacter oxydans*

**DOI:** 10.3390/molecules29122932

**Published:** 2024-06-20

**Authors:** Katarzyna Górska, Zbigniew Garncarek

**Affiliations:** Department of Biotechnology and Food Analysis, Wroclaw University of Economics and Business, 53-345 Wroclaw, Poland; katarzyna.gorska@ue.wroc.pl

**Keywords:** crude glycerol, dihydroxyacetone, *Gluconobacter oxydans*, fed-batch culture

## Abstract

The strain *Gluconobacter oxydans* LMG 1385 was used for the bioconversion of crude glycerol to dihydroxyacetone. The suitability of fed-batch cultures for the production of dihydroxyacetone was determined, and the influence of the pH of the culture medium and the initial concentration of glycerol on maximizing the concentration of dihydroxyacetone and on the yield and speed of obtaining dihydroxyacetone by bioconversion was examined. The feeding strategy of the substrate (crude glycerol) during the process was based on measuring the dissolved oxygen tension of the culture medium. The highest concentration of dihydroxyacetone P_K_ = 175.8 g·L^−1^ and the highest yield Y_P/Sw_ = 94.3% were obtained when the initial concentration of crude glycerol was S_0_ = 70.0 g·L^−1^ and the pH of the substrate was maintained during the process at level 5.0.

## 1. Introduction

The International Energy Agency (IEA) predicts that biodiesel production will reach 39.3 billion liters in 2027 [1]. When producing 10 kg of biodiesel by transesterification of rapeseed oil, 1 kg of glycerol is produced [2]. Such a large amount of crude glycerol creates a problem with its management, mainly since, in addition to pure glycerol, it also contains methanol, mono and diglycerides, free fatty acids, phospholipids, tocopherols, color substances, and soaps [3]. Using crude glycerol for feeding animals is problematic due to its contamination [4]. High viscosity, high flash point, and the presence of water make it an unsuitable alternative to fossil fuels [5,6]. Purification of crude glycerol to obtain a valuable commercial product is a complex and expensive process. Work has been going on for many years to develop various ways of using crude or partially purified glycerol generated in biodiesel production. Its use has been investigated, among others, for the production of erythritol [7], glyceric acid [8], citric acid [9,10], and other bioproducts [11]. None of these methods have found industrial application, and work on using crude glycerol is still ongoing.

One way to manage crude glycerol may be its bioconversion to dihydroxyacetone (DHA), which has various applications, e.g., as an ingredient of self-tanning creams [12], as a component of biomaterials that stops hemorrhages, and in the treatment of vitiligo [13,14], as a sweetener and dietary supplement [15,16] and an emulsifier and plasticizer [17,18]. Recently, several researchers have been working on using crude glycerol as a raw material for obtaining DHA [19,20,21,22,23]. Some used immobilized cells or cell extracts for this purpose [21,23,24,25]. Bioconversion of pure glycerol to DHA using bacteria requires maintaining appropriate conditions, especially regarding the glycerol concentration, to limit substrate inhibition. Few teams have dealt with obtaining DHA through fed-batch culture. It is difficult to determine the moment of introducing the next portion of raw material into the bioreactor.

Our research aimed to use crude glycerol to obtain DHA using unmodified *Gluconobacter oxydans* LMG 1385. In order to reduce the inhibitory effect of high substrate and product concentrations on the bioconversion of glycerol to DHA, fed-batch cultivation was used. The fundamental problem in these cultures is determining the strategy of feeding the substrate to the medium during the culture. The literature on the subject has yet to find an automatic method of feeding glycerol into the bioreactor. We developed an automatic method of feeding glycerol during cultivation. It was based on the fact that oxygen dissolved in the culture medium is used to oxidize glycerol. Each portion of crude glycerol was added automatically as the culture medium saturation with dissolved oxygen increased. Feeding stopped when the dissolved oxygen tension (DOT) in the culture medium began to increase, and the maximum dose of glycerol was limited to limit possible substrate inhibition.

## 2. Results

### 2.1. The Influence of the pH of the Culture Medium on DHA Production

In fed-batch cultures of *Gluconobacter oxydans* LMG 1835 carried out in a bioreactor, the influence of pH regulation of the culture medium on the speed and efficiency of obtaining DHA by bioconversion and the concentration of bacterial biomass was determined. For this purpose, a series of fed-batch cultures were carried out in culture media with a pH ranging from 4.0 to 6.0. Cultures with variable pH were also performed. Based on the literature data [26], the optimal pH for the growth of the biomass of *Gluconobacter oxydans* LMG 1385 was used at the level of 6.0 and was changed after 24 h of culture to the optimal pH for the bioconversion of waste glycerol to DHA at the level of 5.0.

Comparison of the total biomass growth rate (R_X_) and bioconversion of crude glycerol to DHA (R_P_), the total yield of DHA by bioconversion concerning the introduced substrate (Y_P/Sw_), the final biomass concentration (X_K_) and the final DHA (P_K_) concentrations in fed-batch cultures of *Gluconobacter oxydans* LMG 1385 carried out in culture media with pH adjusted at various levels are presented in Table 1.

The data analysis in Table 1 shows that the highest increase in the *Gluconobacter oxydans* LMG 1385 biomass occurred in a fed-batch culture with a pH of 5.0 and amounted to 1.60 g·L^−1^. In the remaining cultures, bacterial biomass (X) concentration was lower and ranged from 1.38 to 1.51 g·L^−1^. The volumetric biomass growth rate (R_X_) for all fed-batch cultures with pH-controlled ranged from 0.02 to 0.03 g·L^−1^·h^−1^.

The highest final DHA concentration (P_K_), 175.8 g·L^−1^, was found in the fed-batch culture with a pH adjusted at 5.0. The highest DHA yield about the introduced glycerol and the highest DHA production efficiency coefficient value, K_ef_ = 2.4 g·L^−1^·h^−1^, were also obtained at this pH.

The lowest final product concentration was obtained in fed-batch culture with a pH adjusted to 6.0, amounting to 91.6 g·L^−1^. The final product concentration ranged from 118.9 to 138.8 g·L^−1^ in the remaining fed-batch cultures.

The highest volumetric bioconversion rate of crude glycerol to DHA (R_P_) was obtained in a fed-batch culture with a pH adjusted at 5.0 (R_P_ = 2.55 g·L^−1^·h^−1^). The lowest volumetric rate of bioconversion of crude glycerol to DHA was observed in the culture with pH adjusted at 6.0 (R_P_ = 1.68 g·L^−1^·h^−1^).

In the case of culture with a change in the pH of the culture medium after 24 h from 6.0 to 5.0, the expected results were not obtained. Hu and coworkers [26,27] provide two optimal pH values of the culture medium: pH = 6.0 for the growth of the biomass of the bacteria *Gluconobacter oxydans* ZJB11001 and pH = 5.0, which allows to optimize the process of bioconversion of glycerol to DHA. In the case of the fed-batch culture of the strain *Gluconobacter oxydans* LMG 1385, despite obtaining a high concentration of bacterial biomass (X_K_ = 1.51 g·L^−1^), the final DHA concentration was equal to P_K_ = 138.8 g·L^−1^ and lower by 21% than in a culture in which the pH of the culture medium was regulated at 5.0 throughout the culture period.

**Figure 1:**
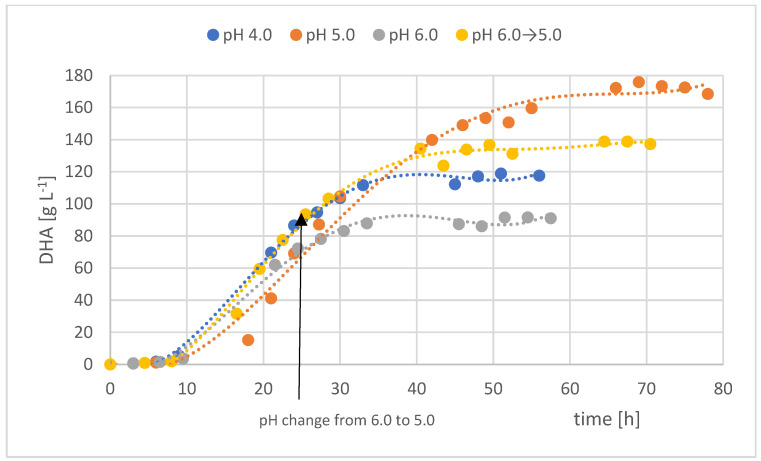
The course of changes in DHA concentration in fed-batch cultures in pH-regulated culture media.

**Figure 2:**
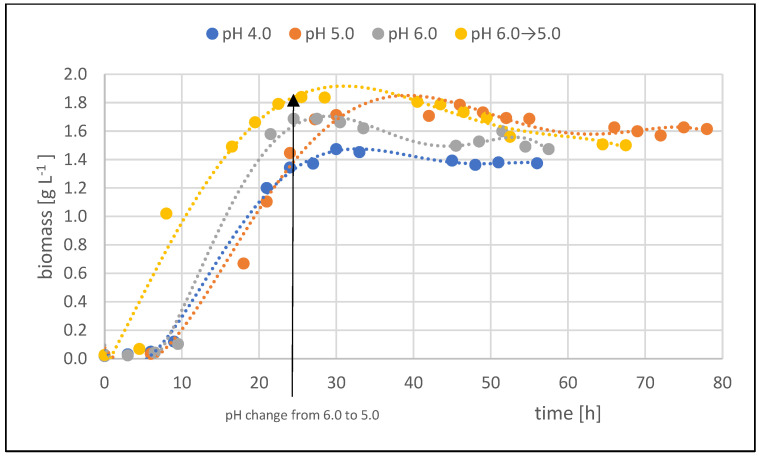
Changes in biomass concentration fed-batch cultures in culture media with adjusted pH.

Figure 1 shows the development of DHA concentration over time in individual fed-batch with different pH. Intensive bioconversion of crude glycerol to DHA began on the second day of the process, after the phase of intensive bacterial biomass multiplication and initiation of crude glycerol bioconversion (Figure 2). Changes in DHA concentration over time had a similar course.

Due to the results obtained and the kinetic parameters characterizing individual fed-batch cultures, in further studies on the production of DHA by bioconversion in fed-batch cultures of *Gluconobacter oxydans* LMG 1835, the pH of the culture medium was equal to 5.0.

### 2.2. The Influence of Crude Glycerol Concentration on the Efficiency of Obtaining Dihydroxyacetone by Bioconversion

In studies on the influence of the initial concentration of glycerol in the culture medium on the process of obtaining DHA by bioconversion of the strain *Gluconobacter oxydans* LMG 1385 in fed-batch cultures, the following initial concentrations of crude glycerol [g·L^−1^] were used: 40.0; 55.0; 70.0; 85.0; 100.0. The analysis of the course of fed-batch cultures showed that the earliest moment to start feeding the culture with waste glycerol is the 15th and 20th hour of the process in the case of cultures with a low initial concentration of crude glycerol in the culture medium (S_0_ = 40.0 g·L^−1^ and S_0_ = 55.0 g·L^−1^) and the end of the first day of the process in the case of other cultures with a higher initial concentration of crude glycerol (S_0_ = 70.0; 85.0 and 100.0 g·L^−1^). At that time, there was a sufficiently high biomass concentration in the culture medium, and the bioconversion of crude glycerol to dihydroxyacetone proceeded at a high rate.

The results of research on the influence of the initial concentration of crude glycerol in the culture medium on the development of basic kinetic parameters characterizing the growth of biomass, consumption of crude glycerol, and obtaining of DHA by bioconversion in fed-batch cultures of *Gluconobacter oxydans* LMG 1385 carried out in a bioreactor, in a culture media with different initial concentrations of waste glycerol are presented in Table 2.

The analysis of the test results presented in Table 2 showed that in the fed-batch culture of *Gluconobacter oxydans* LMG 1385 in a culture medium with a glycerol concentration of 70.0 g·L^−1^, the highest final DHA concentration (P_K_) was obtained, equal to 175.8 g·L^−1^, with the final biomass concentration of X_K_ = 1.60 g·L^−1^. Moreover, this variant achieved the highest yield of obtaining DHA by bioconversion (Y_P/Sw_ = 94.3%) and the highest DHA production efficiency coefficient (K_ef_ = 2.4 g·L^−1^·h^−1^). The average volumetric rate (R_P_) of bioconversion of crude glycerol to DHA was worse than in the other cultures because the maximum concentration of DHA in the culture with the initial concentration of crude glycerol at the level of 70.0 g·L^−1^ was achieved in a longer time (t = 69 h). The use of lower or higher initial concentrations of crude glycerol resulted in a decrease in the final DHA concentration (P_K_) and the efficiency (K_ef_) and total yield (Y_P/Sw_) of obtaining DHA by bioconversion.

**Figure 3:**
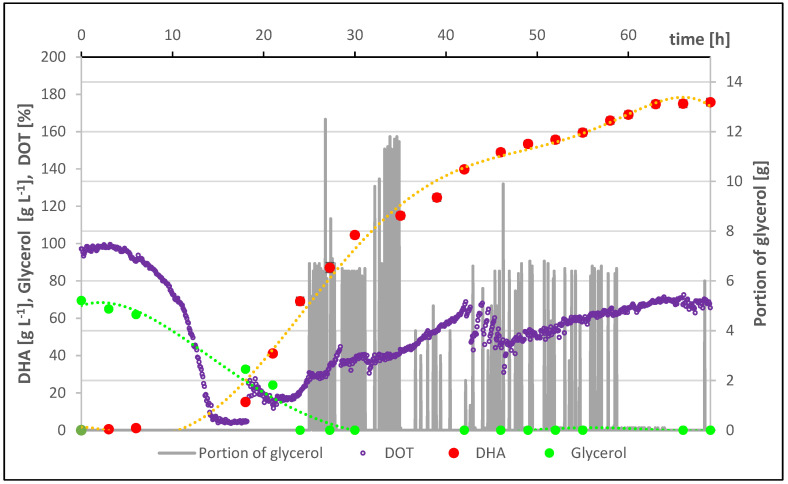
The course of fed-batch cultivation of *Gluconobacter oxydans* LMG 1385 in a culture medium with an initial concentration of waste glycerol of 70.0 g·L^−1^.

The course of the best fed-batch culture of the bacteria *Gluconobacter oxydans* LMG 1385 in a culture medium with an initial concentration of crude glycerol of 70.0 g·L^−1^ carried out in a bioreactor is shown in Figure 3.

The feeding of the culture with crude glycerol began after 25 h of culture when the oxygen saturation level of the culture medium began and reached 25%. The culture was fed with small portions of waste glycerol (maximum 5 g·L^−1^), and the dosing was stopped when the oxygen saturation level began to drop. In the initial period of feeding the culture, quick responses from the system were obtained, and the subsequent dosing of waste glycerol took place at short intervals. The analysis of the culture course presented in Figure 3 shows that the DHA production process can be divided into three stages: multiplication of bacterial biomass, initiation of bioconversion of waste glycerol to DHA, and intensive bioconversion of waste glycerol to DHA.

A comparison of the results obtained in fed-batch cultures with the results of traditional submersible batch cultures showed that higher final DHA (PK) concentrations were obtained in fed-batch cultures than in traditional batch cultures.

## 3. Discussion

The process of bioconversion of glycerol to DHA by submerged batch culture is influenced by many factors, e.g., the ability of the selected strain of acetic bacteria to bioconvert crude glycerol to DHA, the composition of the culture medium, and the conditions of the process.

In our research, crude glycerol from the production of biofuels was used as the only substrate for obtaining DHA by bioconversion, considering it as a full-value raw material for the production of DHA using the *Gluconobacter oxydans* LMG 1385. It was simultaneously consumed as a carbon source by *Gluconobacter oxydans* LMG 1385. Unlike other authors, no additional carbon sources were used, such as glucose, maltose, mannitol [28], and sorbitol [20,28,29,30,31], to increase the growth of acetic bacteria. Exciting results for obtaining DHA were obtained by Ripoll et al. [32], who investigated several strategies for the immobilization of *Gluconobacter oxydans* NBRC 14819 and the bioconversion of crude glycerol to DHA. The potential production of DHA by bacteria immobilized on the agar matrix was 202.04 g per 1 L after eight uses.

It should be noted, however, that only some authors have researched obtaining DHA by bioconversion using crude glycerol [19,20,21,22,23,32,33]. Therefore, the results of the research conducted as part of this work were also compared with the results of research on the bioconversion of pure glycerol to DHA in fed-batch cultures of acetic bacteria.

Our work optimized the initial concentration of crude glycerol in the culture medium in fed-batch cultures. Most authors dealing with obtaining DHA by bioconversion from pure and raw glycerin in fed-batch cultures did not conduct such experiments. They assumed the initial glycerol concentration at S_0_ = 20 g·L^−1^ [19,26,27,34] or S_0_ = 60 g·L^−1^ [22]. 

In our research, five levels of initial concentration of crude glycerol were tested, ranging from 40.0 to 100.0 g·L^−1^. By examining the influence of the initial concentration of crude glycerol in the culture medium on the efficiency of bioconversion to DHA in fed-batch cultures of *Gluconobacter oxydans* LMG 1385 conducted in a bioreactor, it was found that the optimal initial concentration of crude glycerol in the culture medium should be S_0_ = 70.0 g·L^−1^ because it allows for achieving the highest final concentration of DHA, P_K_ = 175.8 g·L^−1^. Zheng et al.’s [22] studies of fed-batch cultures of *Gluconobacter frateurii* CGMCC 5397 conducted in culture media with crude glycerol obtained a final concentration of DHA at a similar level (P_K_ = 175.44 g·L^−1^) and a production yield that was more than 5% lower (Y_P/Sw_ = 89%) than the authors of this work. Different results from those obtained in this study can be explained using a different, newly isolated species of bacteria (*Gluconobacter frateurii* CGMCC 5397) and different culture conditions. 

A higher optimal initial glycerol concentration in fed-batch culture was determined by Zeng et al. [35]. It was 120 g·L^−1^. However, they used a modified strain of *G. oxydans* WSH-003–4 by knocking out individual dehydrogenase genes unrelated to DHA synthesis.

The research results presented in our study suggest that the initial concentration of glycerol of 70.0 g·L^−1^ in the culture medium does not inhibit the metabolic activity of the bacterium *Gluconobacter oxydans* LMG 1385 due to the high initial concentration of the substrate.

The proper pH of the culture medium is essential for cultivating all microorganisms. Our research tested four pH levels of the culture medium (4.0, 5.0, 6.0, 6.0 → 5.0). In fed-batch cultures of *Gluconobacter oxydans* LMG 1385, carried out in a bioreactor, the pH of the culture medium maintained at 5.0 allowed to obtain the highest final concentration of DHA, P_K_ = 175.8 g·L^−1^, and the production efficiency factor, K_ef_ = 2.4 g·L^−1^·h^−1^.

Hu and co-workers [26,27] provide two optimal pH values for the culture medium: pH = 6.0 for the growth of the biomass of *Gluconobacter oxydans*, and pH = 5.0, which allows optimizing the process of bioconversion of glycerol to DHA. The results of the research carried out as part of this study did not confirm these literature reports because in a fed-batch culture of *Gluconobacter oxydans* LMG 1385 with a change in the pH of the culture medium after 24 h of culture from 6.0 to 5.0, the expected results were not obtained. Despite obtaining a high concentration of bacterial biomass (X_K_ = 1.51 g·L^−1^), the final concentration of DHA was equal to P_K_ = 138.8 g·L^−1^ and lower by 21% than in the fed-batch culture, in which the pH of the culture medium was regulated at 5.0 throughout the entire process.

The choice of the *Gluconobacter oxydans* cultivation method plays a fundamental role in the speed and efficiency of the DHA bioconversion process. The analysis of the world literature showed that most authors, in their studies on the bioconversion of glycerol to DHA using acetic bacteria, used submerged batch cultures carried out in flasks on a shaker [19,22,36] and in a bioreactor [19,20,27,37,38,39]. Some researchers, in order to eliminate the disadvantages of submerged batch cultures, primarily the phenomenon of substrate inhibition, conducted fed-batch cultures of *Gluconobacter oxydans* in a bioreactor [19,22,26,27,31,34,40,41].

In the fed-batch cultures of *Gluconobacter oxydans* LMG 1385 presented in our research, the level of oxygen saturation of the culture medium was used as a criterion for feeding the culture with a fresh portion of crude glycerol. An increase in oxygen saturation of the culture medium above the set level was a signal to introduce a new portion of crude glycerol. Feeding was stopped when the oxygen saturation of the culture medium began to decrease, which meant that the introduced glycerol was oxidized to DHA. During the process, the level of oxygen saturation of the culture medium, used as a dosing criterion, was not kept constant but had to be increased so that the entire system responded to introducing a fresh portion of the substrate. Other authors also observed an increase in the level of oxygen saturation of the culture medium during the oxidation phase of glycerol to DHA [22]. The method used allowed for obtaining a high final concentration of DHA (P_K_ = 175.8 g·L^−1^), a high yield of DHA (Y_P/Sw_ = 94.3%) about the introduced substrate, and, at the same time, to maintain a low concentration of glycerol in the culture medium. In our research conducted using this method, a higher concentration of DHA was obtained compared to the results of research by some other authors conducting fed-batch cultures of *Gluconobacter oxydans* and using both crude and pure glycerol as a substrate: 165.5 g·L^−1^ [34]; 156.3 g·L^−1^ [42].

In work on the bioconversion of pure glycerol to DHA in fed-batch cultures, Hu et al. [27] obtained a higher final product concentration, P_K_ = 209.6 g·L^−1^. However, despite the use of pure glycerol as a substrate and the use of the *Gluconobacter oxydans* strain mutated using UV radiation, the obtained efficiency of the bioconversion process at the level of Y_P/Sw_ = 90.1% was lower than that obtained in our work.

Using fed-batch culture with an initial glycerol concentration of 120 g·L^−1^ and six feedings with 20 g·L^−1^ portions of glycerol, Zeng et al. [35], obtained a high DHA concentration of 198.81 g·L^−1^. However, the authors used a modified strain of *G. oxydans* WSH-003–4 by knocking out individual dehydrogenase genes unrelated to DHA synthesis. Moreover, they did not use an automatic method of determining the moment of dosing the next glycerol portion. It allowed for six additional feedings and the obtained glycerol conversion rate of 82.84%. In our research, using an automatic method of determining the moment of introducing the next portion of glycerol, based on measuring the degree of dissolved oxygen saturation of the culture medium, we achieved a much higher yield of obtaining DHA about the introduced glycerol, amounting to 94.3%.

Liu and coworkers [19] also conducted a fed-batch culture with crude glycerol. However, they used a newly isolated strain of *Gluconobacter frateurii* CGMCC 5397. During the process, they kept the crude glycerol concentration low (from 5 to 15 g·L^−1^). The effect was faster growth of bacterial cells and an extension of the stationary phase by limiting substrate inhibition. They obtained a much lower final concentration of DHA (P_K_ = 125.8 g·L^−1^) and a lower yield about the glycerol used (Y_P/S_ = 90.5%) compared to the test results obtained in our work. In their later studies, the same team, using a similar substrate feeding strategy, conducted fed-batch cultures of *Gluconobacter frateurii* CGMCC 5397 in a 30 L bioreactor under conditions ensuring a high value of the oxygen transfer coefficient of k_La_ = 82.14 h^−1^ [22]. The feeding strategy was to regulate the feeding rate every 2 h to maintain the crude glycerol concentration in the culture medium from 5 to 25 g·L^−1^. At the initial concentration of waste glycerol S_0_ = 60 g·L^−1^ and the pH of the culture medium at 6.0, the team obtained lower substrate yield (Y_P/Sw_ = 89%) and a similar final concentration of DHA (P_K_ = 175.44 g·L^−1^) as in our study.

A similar strategy of conducting fed-batch culture for the bioconversion of glycerol to DHA as in our work was used by Hu and coworkers [26]. The difference was that Hu’s team used a constant oxygen saturation level of 30% in the culture medium as a criterion for introducing a new portion of glycerol. In fed-batch cultures of *Gluconobacter oxydans* ZJB09112, Hu et al. used pure glycerol as the substrate. This method of feeding the culture medium and the different pH levels of the medium used for bacterial growth (pH = 6.0) and for the bioconversion of glycerol to DHA (pH = 5.0) allowed to obtain a similar final concentration of DHA (P_K_ 175.9 g·L^−1^) as in our work, and process yield was lower by over 7% (Y_P/Sw_ = 87%).

The same team used pulsed feeding of the culture medium with pure glycerol, taking the consumption time and the concentration of the remaining glycerol as the criteria. In the culture in which pure glycerol was fed four times, the final concentration of DHA was achieved, P_K_ = 161.9 g·L^−1^, the volumetric bioconversion rate of glycerol to DHA R_P_ = 2.38 g·L^−1^·h^−1^ and the product yield at the level of Y_P/Sw_ = 88.7% [34]. Hu and coworkers [42], using the same strategy of feeding at the culture of *Gluconobacter oxydans* ZJB09113 in an airlift bioreactor, obtained the final concentration of DHA P_K_ = 156.3 g·L^−1^ and the volumetric bioconversion rate of glycerol to DHA at the level of R_P_ = 2.17 g·L^−1^·h^−1^.

Bauer [29] used a similar feeding strategy based on a constant glycerol concentration in the culture medium ranging from 5 to 25 g·L^−1^. However, the authors carried out repeated two-stage fed-batch cultures of *Gluconobacter oxydans* and obtained the final concentration of DHA P_K_ = 162 g·L^−1^ after 170 h of the process. Bauer [41] also reports that using a semi-continuous two-stage repeated fed-batch process with a combination of a laboratory-scale bubble column and a laboratory-scale stirred reactor obtained a final concentration of DHA of 220 kg·m^−3^ during a two-stage process. It was possible thanks to the fact that intact membrane-bound glycerol-oxidizing dehydrogenase was still active both in the irreversibly growth-inhibited cells and in the cell debris.

The highest final concentration of DHA obtained in our research was 16,1% lower than the results obtained by Hu and Zheng [27] in a fed-batch submerged culture with the mutant strain of *Gluconobacter oxydans*. However, the authors used pure glycerol as a substrate and different pH levels of the culture medium for bacterial growth (pH = 6.0) and glycerol for DHA bioconversion (pH = 5.0). They also controlled the oxygen saturation of the culture medium, gradually increasing its level every 10% (in the range from 20 to 40% saturation) and maintaining a constant glycerol concentration (in the range from 5 to 10 g·L^−1^). They used a substrate-feeding method based on the predicted glycerol consumption time. They found that the bioconversion process of glycerol to DHA can be divided into three stages that differ in oxygen demand. In the first stage of the process, the oxygen saturation of the culture medium was maintained at 20% for 24 h to ensure the appropriate concentration of dissolved oxygen for the growth of bacterial cells. In this step, glycerol was consumed both as a carbon source and as a substrate in the bioconversion. In the second stage of cultivation, they increased the oxygen saturation level of the culture medium to 30% and maintained it until 60 h of the process. During this stage, cell growth almost wholly ceased, and glycerol was mainly used as a substrate for the bioconversion of DHA. In the last stage, after 60 h of the process, when the concentration of DHA was higher than 150 g·L^−1^, they increased the oxygen saturation level of the culture medium to 40%. At this stage, the production rate of DHA decreased, probably due to the high concentration of DHA, which inhibits the glycerol bioconversion process [27].

## 4. Materials and Methods

### 4.1. Bacterial Strain and Culture Media

The research used the strain *Gluconobacter oxydans* LMG 1385 from the Belgian Collection of Microorganisms (Bruxelles, Belgium), isolated by Kondo from dried fruits. A pure culture of the acetic bacteria strain was stored in test tubes on a medium of slants with the composition (in g per liter): D-mannitol 25.0; yeast extract 5.0; peptone 3.0; agar 15.0. Its storage period at 4 °C was one month. After this time, the pure culture was transferred to fresh slants.

### 4.2. Crude Glycerol

Crude glycerol, the main by-product of the transesterification reaction, originating from Wratislavia-BIO Sp. (Wrocław, Poland), was used to prepare culture media. Its characteristics are presented in Table 3.

Organic residue MONG (Matter Organic Non-Glycerol) is defined as 100% minus the sum of the percentages of glycerol, ash, and water.

### 4.3. Preparation of Inoculum

A liquid culture of acetic bacteria at 5% by volume was used as the inoculum. Medium (150 mL) was introduced into conical flat-bottomed flasks with a capacity of 500 mL, and then they were sterilized in an autoclave at 121 °C for 30 min. After removal from the autoclave and cooling to approximately 30 °C, acetic bacteria were introduced into each flask and multiplied on a shaker for 24 h at 30 °C at 200 rpm. During the BIOMER 10 bioreactor tests, the inoculation medium had the same composition as the culture medium.

### 4.4. Bioreactor Process

The research was conducted in the BIOMER 10 laboratory bioreactor, designed and built at our Department of Food Biotechnology.

In studies on the effect of the initial concentration of crude glycerol in fed-batch cultures carried out in a bioreactor, the culture medium contained (in g·L^−1^) crude glycerol at a concentration of 40.0, 55.0, 70.0, 85.0, or 100.0 and yeast extract 3.7. The culture medium was supplemented with water. Sterile crude glycerol was used as the feed medium.

During the research on determining the influence of pH on the efficiency of the bioconversion process of crude glycerol to DHA, a culture medium was used with the following composition (g·L^−1^): crude glycerol 70.0; yeast extract 3.7. The pH of the culture medium was maintained at the levels of 4.0, 5.0, 6.0, and 6.0 (initially) → and then 5.0 in the further stage of culture after bacterial multiplication in fed-batch cultures. The addition of 2 M NaOH regulated the pH of the medium.

In order to set up a fed-batch culture, 80% of the bioreactor capacity was filled with culture medium before starting the culture. After introducing the inoculum, the process was carried out similarly to the batch method. Crude glycerol was introduced into the bioreactor using a peristaltic pump type 371 (UNIPAN, Warszawa, Poland), controlled by a program written using the GENIE 2.12 control and data acquisition system. The amount of crude glycerol introduced depended on the oxygen saturation of the culture medium (pO_2_). The program limited the maximum dose of crude glycerol introduced so that its concentration did not exceed 5 g·L^−1^. The end of culture was considered to be the lack of increase in DHA concentration in the culture medium. The oxygen saturation value of the culture medium, which was used to determine the moment of adding the next portion of the substrate—glycerol, ranged from 30% (initially) to 70% (at the end of the process). This threshold level of oxygen saturation of the substrate was increased gradually. Its determination was related to the observation of the system’s response to adding a new portion of glycerol (i.e., a decrease in oxygen saturation of the substrate after adding another portion of glycerol, which indicated the resumption of the oxidation process of glycerol to DHA.

The regulation of substrate dosage is based on the measurement of oxygen saturation of the culture medium, which is based on changes in the respiratory activity of microorganisms caused by the presence or absence of carbon and energy sources in the medium. After the exhaustion of carbon and energy sources from the culture medium, the respiratory activity of microbial cells decreases (rate of oxygen absorption), which, while maintaining constant oxygenation conditions, causes a gradual increase in oxygen saturation of the culture medium. Conversely, introducing a new portion of substrate into the culture medium increases the vital activity of microorganisms, and thus, the rate of oxygen consumption increases, which is externally expressed by a decrease in oxygen saturation of the culture medium.

### 4.5. Analytical Methods

DHA concentration was determined using colorimetric methods with 3,5-dinitrosalicylic acid [48] and chromatography. In the HPLC method, the Knauer Eurokat H C-42 column was converted from the hydrogen form to the calcium form by rinsing it with 0.25 M Ca(NO_3_)_2_ at a flow rate of 0.2 mL·min^−1^ for 6 h at 60 °C. The HPLC methods for both DHA and glycerol were performed on a liquid chromatograph (Perkin Elmer, Waltham, MA, USA) with a UV–Vis detector and a refractometer [49]. The column was operated at 60 °C. The samples were eluted with water at a 0.6 mL/min flow rate.

The concentration of bacterial biomass was determined spectrophotometrically. A total of 3 mL of culture medium was taken from each sample and centrifuged for 30 min at 5000 rpm in the MPW-251 laboratory centrifuge (MPW MED. INSTRUMENTS, Warsaw, Poland). The supernatant was separated from the sediment, the sediment was suspended in distilled water, and its absorbance was measured at a wavelength of λ = 600 nm. The bacterial biomass concentration was calculated based on the standard curve.

### 4.6. Calculation of Kinetic Parameters of Culture

The volumetric growth rate of bacterial biomass (*R_X_*) in fed-batch cultures was calculated from the following equation:

$$R_{X}=\frac{dX}{dt}\;\;\;\;\;\;\;\;[g\cdot L^{-3}\cdot h^{-1}]$$

Wherein

*dX*—increase in biomass concentration in the culture medium during *dt*, [g·L^−1^],

*dt*—cultivation time, [h].

The volumetric consumption rate of crude glycerol (*R_S_*) in fed-batch cultures was calculated from the following equation:

$$R_{S}=\frac{dS}{dt}\;\;\;\;\;\;\;\;[g\cdot L^{-1}\cdot h^{-1}]$$

Wherein

*dS*—decrease in crude glycerol concentration in the culture medium over time *dt*, [g·L^−1^]. 

The specific rate of crude glycerol consumption (*Q_S_*) in fed-batch cultures was calculated from the following equation:

$$Q_{S}=\frac{dS}{dt\cdot X}\;\;\;\;\;\;\;\;[g\cdot L^{-1}\cdot h^{-1}]$$

The volumetric rate of DHA biosynthesis (*R_P_*) in fed-batch cultures was calculated from the following equation:

$$R_{P}=\frac{dP}{dt}\;\;\;\;\;\;\;\;[g\cdot L^{-1}\cdot h^{-1}]$$

Wherein

*dP*—increase in DHA concentration in the culture medium.

The specific rate of DHA biosynthesis (*Q_P_*) in fed-batch cultures was calculated from the equation:

$$Q_{P}=\frac{R_{P}}{X}=\frac{dP}{dt\cdot X}\;\;\;\;\;\;\;\;[g\cdot g^{-1}\cdot h^{-1}]$$

DHA production yield (*Y_P/S_*) about the introduced crude glycerol in fed-batch cultures was calculated from the following equation:

$$Y_{P/S}=\frac{P_{K}}{S_{0}+\sum S_{Z}}\;\;100\;\;\;\;\;\;\;\;[\%\;(m/m)]$$

Wherein

*P_K_*—final concentration of DHA in the culture medium, [g·L^−1^],

*S_0_*—concentration of crude glycerol in the culture medium at the beginning of the process, [g·L^−1^],

Ʃ*S_Z_*—the amount of crude glycerol added to the culture medium when feeding the culture, [g·L^−1^].

The biomass yield of the introduced crude glycerol (*Y_X/S_*) in fed-batch cultures was calculated from the following equations:

$$Y_{X/S}=\frac{X_{K}}{S_{0}+\sum S_{Z}}\;\;100\;\;\;\;\;\;\;\;\;[\%\;(m/m)]$$

Wherein

*X_K_*—final biomass concentration in the culture medium, [g·L^−1^].

The DHA production efficiency coefficient (*Kef*) was calculated from the following equation [50]:

$$K_{ef}=R_{P}\cdot Y_{P/Sw}\;\;\;\;\;\;\;\;[g\cdot L^{-3}\cdot h^{-1}]$$

*R_P_*—the volumetric rate of DHA production, [g·L^−1^·h^−1^],

*Y_P/S_*—DHA yield coefficient about the crude glycerol introduced, [-]

### 4.7. Statistical Analysis

The determinations were performed in triplicate. Mean values and standard deviation were calculated using Excel 365 MSO v 2403. One-way analysis of variance was performed using the Statistica v. 12 program.

## 5. Conclusions

The research subjects were fed-batch cultures of acetic bacteria in a medium containing crude glycerol from the production of biofuels carried out in a bioreactor. Based on the research conducted, the following conclusions were found:

- Crude glycerol is a valuable raw material for obtaining DHA by bioconversion using the *Gluconobacter oxydans* LMG 1385.
- The pH of the culture medium significantly impacted the efficiency of the DHA production process using the *Gluconobacter oxydans* LMG 1385. The optimal pH of the medium during fed-batch cultures was 5.0.
- The most suitable method for producing DHA from crude glycerol generated in biodiesel production is fed-batch cultivation of the *Gluconobacter oxydans* LMG 1385 with the feed based on the level of oxygen saturation of the culture medium. However, a change in the control value is required during the bioconversion stage.
- In bioconversion of crude glycerol to DHA, three stages can be distinguished: multiplication of bacterial biomass, initiation of glycerol to DHA, and intensive bioconversion of glycerol to DHA.

## Figures and Tables

**Table 1:** The influence of the pH level of the culture medium on the kinetic parameters of obtaining DHA by bioconversion in fed-batch cultures.

Symbols	Unit	Parameters	Set the pH Value of the Culture Medium			
			4.0	5.0	6.0	6.0 → 5.0
Set parameters						
t	h	Cultivation time	51	69	54.5	67.5
S_0_	g·L^−1^	Initial concentration of crude glycerol in the culture medium	70.0	70.0	70.0	70.0
Result parameters						
S_K_	g·L^−1^	The final concentration of crude glycerol in the culture medium	37.8 ± 1.13	0.0	53.1 ± 1.59	31.2 ± 0.93
R_S_	g·L^−1^·h^−1^	Average volumetric consumption rate of crude glycerol	2.90 ± 0.09	2.7 ± 0.080	2.13 ± 0.06	2.72 ± 0.08
X_K_	g·L^−1^	Final biomass concentration in the culture medium	1.38 ± 0.04	1.60 ± 0.05	1.49 ± 0.04	1.51 ± 0.04
R_X_	g·L^−1^·h^−1^	Average volumetric growth rate of biomass	0.03 ± 0.001	0.02 ± 0.001	0.03 ± 0.001	0.02 ± 0.001
P_K_	g·L^−1^	Final DHA concentration in the culture medium	118.9 ± 3.50	175.8 ± 5.20	91.6 ± 2.70	138.8 ± 4.16
R_P_	g·L^−1^·h^−1^	Average volumetric rate of bioconversion of crude glycerol to DHA	2.33 ± 0.07	2.55 ± 0.07	1.68 ± 0.05	2.06 ± 0.06
Q_P_	g·g^−1^·h^−1^	The average specific rate of bioconversion of crude glycerol to DHA	1.69 ± 0.05	1.59 ± 0.04	1.13 ± 0.03	1.37 ± 0.04
Y_X/Sw_	% (*m*/*m*)	Total biomass yield	0.7 ± 0.012	0.9 ± 0.026	0.9 ± 0.027	0.7 + 0.022
Y_P/Sw_	% (*m*/*m*)	Total DHA yield	64.1 ± 1.92	94.3 ± 2.80	54.2 ± 1.62	64.7 ± 1.94
K_ef_	g·L^−1^·h^−1^	DHA production efficiency factor	1.5	2.4	0.9	1.3

The average glycerol feed rate 8.47 ± 3.8 [g·h^−1^].

**Table 2:** Basic kinetic parameters characterizing the production of DHA by bioconversion of crude glycerol in fed-batch cultures of *Gluconobacter oxydans* LMG 1385, with different initial concentrations of crude glycerol in the culture medium.

Symbols	Unit	Parameters	Initial Concentration of Crude Glycerol in Fed-Batch Cultures				
			40.0	55.0	70.0	85.0	100.0
Set parameters							
t	h	Cultivation time	48	44	69	45	69
t_Z_	h	Start time of crude glycerol feeding	15	20	26.75	27	24
Result parameters							
S_K_	g·L^−1^	The final concentration of crude glycerol in the culture medium	33.4 ± 1.00	14.3 ± 0.41	0.0	3.2 ± 0.09	7.8 ± 0.23
R_S_	g·L^−1^·h^−1^	Average volumetric consumption rate of crude glycerol	3.42 ± 0.10	3.26 ± 0.09	2.70 ± 0.08	3.20 ± 0.09	2.68 ± 0.08
Q_S_	g·g^−1^·h^−1^	Average specific consumption rate of crude glycerol	1.99 ± 0.05	1.82 ± 0.05	1.69 ± 0.05	2.31 ± 0.06	1.85 ± 0.05
X_K_	g·dm^−1^	Final biomass concentration in the culture medium	1.72 ± 0.05	1.79 ± 0.05	1.60 ± 0.04	1.38 ± 0.04	1.45 ± 0.04
R_X_	g·L^−1^·h^−1^	Average volumetric growth rate of biomass	0.04 ± 0.001	0.04 ± 0.001	0.02 ± 0.001	0.03 ± 0.001	0.02 ± 0.001
P_K_	g·L^−1^	Final DHA concentration in the culture medium	125.3 ± 3.75	123.1 ± 3.68	175.8 ± 5.20	123.0 ± 3.67	145.5 ± 4.35
R_P_	g·L^−1^·h^−1^	Average volumetric rate of bioconversion of crude glycerol to DHA	2.61 ± 0.07	2.80 ± 0.08	2.55 ± 0.07	2.73 ± 0.08	2.11 ± 0.06
Q_P_	g·g^−1^·h^−1^	The average specific rate of bioconversion of crude glycerol to DHA	1.52 ± 0.04	1.56 ± 0.04	1.59 ± 0.04	1.98 ± 0.05	1.46 ± 0.04
Y_X/Sw_	% (*m*/*m*)	Total biomass yield	0.8 ± 0.02	1.1 ± 0.03	0.9 ± 0.02	0.9 ± 0.02	0.8 ± 0.02
Y_P/Sw_	% (*m*/*m*)	Total DHA yield	63.4 ± 1.90	78.1 ± 2.31	94.3 ± 2.80	83.6 ± 2.50	75.6 ± 2.26
K_ef_	g·L^−1^·h^−1^	DHA production efficiency factor	1.7	2.2	2.4	2.3	1.6

**Table 3:** Characteristics of crude glycerol.

No.	Component	Unit	Quantity	Test Methods
1.	Glycerol	% (*m*/*m*)	84.5	[43]
2.	Methanol	% (*m*/*m*)	0.01	[44]
3.	M O. N. G.	% (*m*/*m*)	1.56	[45]
4.	Ash as NaCl	% (*m*/*m*)	6.74	[46]
5.	Water	% (*m*/*m*)	7.2	[47]

## Data Availability

Data related to this study is available from the corresponding author upon reasonable request.
